# Distinct prognostic values of S100 mRNA expression in breast cancer

**DOI:** 10.1038/srep39786

**Published:** 2017-01-04

**Authors:** Shizhen Zhang, Zhen Wang, Weiwei Liu, Rui Lei, Jinlan Shan, Ling Li, Xiaochen Wang

**Affiliations:** 1 Department of Surgical Oncology, Second Affiliated Hospital, Zhejiang University School of Medicine, No. 88, Jiefang Road, Hangzhou, Zhejiang 310009, China; 2 Cancer Institute (Key Laboratory of Cancer Prevention & Intervention, National Ministry of Education, Provincial Key Laboratory of Molecular Biology in Medical Sciences), Second Affiliated Hospital, Zhejiang University School of Medicine, No. 88, Jiefang Road, Hangzhou, Zhejiang 310009, China; 3 Department of Laboratory Medicine, Second Affiliated Hospital, Zhejiang University School of Medicine, No. 88, Jiefang Road, Hangzhou, Zhejiang 310009, China; 4 Department of Plastic Surgery, First Affiliated Hospital, Zhejiang University School of Medicine, No. 79, Qingchun Road, Hangzhou, Zhejiang 310009, China; 5 Division of Hematopoietic Stem Cell and Leukemia Research, Beckman Research Institute, City of Hope National Medical Center, Duarte, CA, USA

## Abstract

S100 family genes encode low molecular weight, acidic-Ca^2+^ binding proteins implicating in a wide spectrum of biological processes. S100 family contains at least 20 members, most of which are frequently dysregulated in human malignancies including breast cancer. However, the prognostic roles of each individual S100, especially the mRNA level, in breast cancer patients remain elusive. In the current study, we used “The Kaplan-Meier plotter” (KM plotter) database to investigate the prognostic values of S100 mRNA expression in breast cancer. Our results indicated that high mRNA expression of S100A8, S100A9, S100A11 and S100P were found to be significantly correlated to worse outcome, while S100A1 and S100A6 were associated with better prognosis in all breast cancer patients. We further assessed the prognostic value of S100 in different intrinsic subtypes and clinicopathological features of breast cancer. The associated results will elucidate the role of S100 in breast cancer and may further lead the research to explore the S100-targeting reagents for treating breast cancer patients.

Breast cancer is a lethal disease that leads to 15% of cancer deaths in females worldwide in 2015^1^. Although the incidence and mortality rates are decreasing due to the progresses achieved in screening, diagnostic and treatment modalities, the incidence of breast cancer is increasing, tumor recurrence and metastatic relapse is still the major problem contributing to high death rate^2^. Thus, novel targets that can be used to predict or treat breast cancer are awaiting to explore.

S100 family members are small, acidic-Ca^2+^ binding proteins involving in a wide spectrum of biological processes, of which the first member was discovered in 1965^3^. Now, at least 20 members of S100 family have been identified^4^. The so-called S100 alludes to the solubility in 100% saturated ammonium sulfate at neutral pH. There are five genomic loci encoded S100 proteins: S100B on chromosome 21q22, S100G on the Xp22 chromosome, S100P on chromosome 4p16 and S100Z on chromosome 5q14. The remaining members(S100A1-S100A14, S100A7A and S100A16) are coded in two tandem clusters on chromosome locus 1q21^5^,^6^. Dysregulation of S100 expression is a common occurrence in several human tumors^5^. The expression of S100 proteins display a distinctive pattern in cancers that can be both stage-specific and subtype-specific. For example, S100A2 plays a tumor-suppress role in oral cancer, but as a tumor promoter in lung cancer^7^,^8^. S100A7 functions differing effects in breast cancer depending on the different estrogen receptor(ER) status^9^. Apart from S100A7, other S100 family members, including S100A1, S100A4, S100A6, S100A8, S100A9, S100A11, S100A14, S10016 and S100P, have been reported to express in breast cancer^10^,^11^,^12^,^13^,^14^,^15^,^16^,^17^,^18^. Furthermore, the expression of S100A4, S100A9 S100A14, S100A16 and S100P detected by immunohistochemistry were associated with shorter survival in breast cancer patients^18^,^19^,^20^,^21^. Mckieman *et al*. studied 16 members of S100 gene expression (S100A1-S100A14, S100P and S100B) in breast cancer, only S100A11 and S100A14 were related to poor outcome^22^. Unlike the majority of the S100 family, S100A2 was considered as a tumor suppressor which is down-regulated in breast cancers^23^,^24^. Nevertheless, some S100 family members, for example S100A1, S100A13 or S100G have been rarely studied in breast cancer. The prognostic roles of each individual S100, especially at the mRNA level in breast cancers are still elusive.

KM plotter database was generated using gene expression data and survival information downloaded from GEO(http://www.ncbi.nlm.nih.gov/geo/). Currently, in that database, 3557 patients have relapsed free survival (RFS) data, 1610 have distant metastasis free survival (DMFS) data and 1117 have overall survival (OS) data^25^. It has been widely used to analyze the clinical impact of individual genes to RFS, DMFS and OS of cancers, including lung cancer, breast cancer, ovarian cancer and gastric cancer^26^,^27^,^28^. In this study, we assessed the prognostic role of each member of S100 mRNA expression in human breast cancer patients by KM plotter database.

## Material and Methods

The correlation of individual S100 family members mRNA expression to OS was analyzed on an online database, which was established using gene expression data and survival information of breast cancer patients downloaded from Gene Expression Omnibus (GEO)^25^. Clinical data including ER, PR, HER2 status, lymph node status, differentiation grade, intrinsic subtype and TP53 status were collected. Briefly, 20 individual members of S100 family were entered into the database (http://kmplot.com/analysis/index.php? p=service&cancer=breast) respectively and analyzed with setting different clinical parameters. Then, Kaplan-Meier survival plots with the number-at-risk indicated below, hazard ratio (HR), 95% confidence intervals (CI) and log rank P were obtained on the webpage. P value of <0.05 was considered to be statistically significant.

## Results

### Prognostic values of S100 members in all breast cancer patients

**Figure 1:**
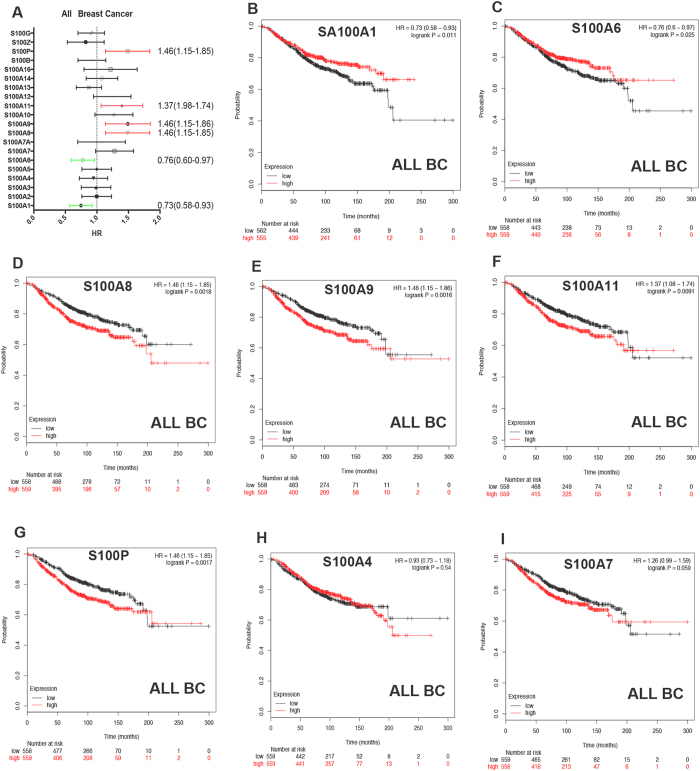
The prognostic value of the mRNA expression of S100A in www.kmplot.com. (**A**) Prognostic HRs of individual S100 members in all breast cancer. (**B–I**) Survival curves of S100A1(the desired Affymetrix IDs is valid: 205334_at), S100A6(Affymetrix IDs: 217728_at), S100A8(Affymetrix IDs: 202917_s_at), S100A9(Affymetrix IDs: 203535_at), S100A11(Affymetrix IDs: 200660_at), S100AP(Affymetrix IDs: 209686_at), S100A4(203186_s_at), and S100A7(205916_at) are plotted for all patients (n = 1117).

We respectively examined the prognostic values of the mRNA expression of twenty S100 family members in breast cancer patients in www.kmplot.com. Among all of them, 6 members were significantly associated with prognosis for all breast cancer patients (Fig. 1A). The survival curves were shown in Fig. 1B–I, we observed S100A1 and S100A6, high mRNA expression were associated with better prognosis (Fig. 1B and C, HR = 0.73, 95% CI: 0.58–0.93, *p* = 0.011 and HR = 0.76, 95% CI: 0.60–0.97, *p* = 0.0246). High mRNA expression of S100A8, S100A9, S100A11 and S100P were significantly associated with worse OS (Fig. 1D–G, HR = 1.46, 95% CI: 1.15–1.85, *p* = 0.0018, HR = 1.46, 95% CI: 1.15–1.86, *p* = 0.0016, HR = 1.37, 95% CI: 1.08–1.74, *p* = 0.0091 and HR = 1.46, 95% CI: 1.15–1.85, *p* = 0.0017 respectively). However, S100A4 was not correlated with OS (Fig. 1H, HR = 0.93, 95% CI: 0.73–1.18, *p* = 0.5414). The mRNA expression levels of the other S100 family members were not correlated with OS (Supplement Fig. 1), although the mRNA expression of S100A7 (Fig. 1I, HR = 1.26 95%CI: 0.99–1.59, *p* = 0.0578) was modestly associated with poor survival.

### Prognostic values of S100 members in different breast cancer subtypes

**Figure 2:**
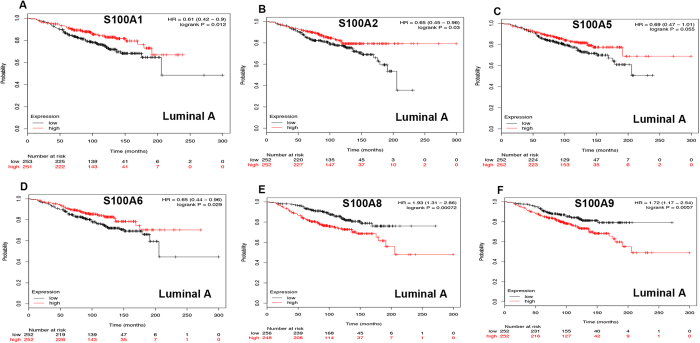
Survival curves of S100A1 (**A**) the desired Affymetrix IDs is valid: 205334_at), S100A2 (**B**) Affymetrix IDs: 204268_at), S100A5 (**C**) Affymetrix IDs: 207763_at), S100A6 (**D**) Affymetrix IDs: 217728_at), S100A8 (**E**) Affymetrix IDs: 202917_s_at) and S100A9 (**F**) Affymetrix IDs: 203535_at) are plotted for luminal A type breast cancer patients (n = 504).

Next, we assessed the prognostic values of S100 family members in breast cancer with different intrinsic subtypes, including luminal A, luminal B, HER2-overexpressing and basal-like. As shown in Fig. 2, for S100A8 (Fig. 2E: HR = 1.93, 95%CI: 1.31–2.86, *p* = 0.0007) and S100A9 (Fig. 2F: HR = 1.72, 95%CI: 1.17–2.54, *p* = 0.0057), high mRNA expression of those S100A members were correlated to lower OS in luminal A type breast cancer patients. For S100A1 (Fig. 2A: HR = 0.61, 95%CI: 0.42–0.90, *p* = 0.0123), S100A2 (Fig. 2B: HR = 0.65, 95%CI: 0.45–0.96, *p* = 0.0297) and S100A6 (Fig. 2D: HR = 0.65, 95%CI: 0.44–0.96, *p* = 0.0288), their mRNA expression levels were associated with longer OS in luminal A type cancers. S100A5 (Fig. 2C: HR = 0.69, 95%CI: 0.47–1.01, *p* = 0.0549) was only modestly associated with better OS but without statistical difference. The rest of S100 members were not related with prognosis in luminal A breast cancer (Supplement Fig. 2).

**Figure 3:**
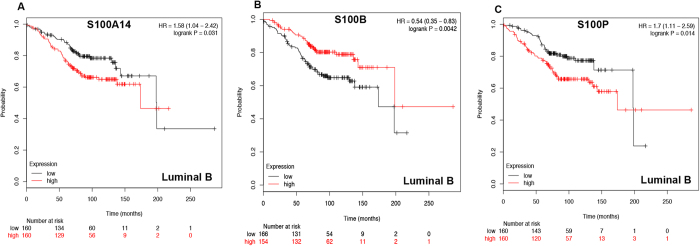
Survival curves of S100A14 (**A**) the desired Affymetrix IDs is valid: 218677_at), S100AB (**B**) Affymetrix IDs: 209686_at) and S100AP (**C**) Affymetrix IDs: 204351_at) are plotted for luminal B type breast cancer patients (n = 320).

In luminal B type breast cancer, S100A14 (Fig. 3A: HR = 1.58, 95%CI: 1.04–2.42, *p* = 0.0313) and S100P (Fig. 3C: HR = 1.7, 95%CI: 1.11–2.59, *p* = 0.014) was correlated to worse OS, however, S100B (Fig. 3B: HR = 0.54 95%CI: 0.35–0.83, *p* = 0.0042) was associated with better prognosis. The rest members of S100 were not correlated to prognosis in luminal B breast cancer (Supplement Fig. 3).

In HER2-overexpressing breast cancer patients, none of high mRNA expression levels of S100 family members were correlated with OS (Supplement Fig. 4). The expression of S100B (Supplement Fig. 4 Q: HR = 0.49, 95%CI: 0.22–1.08, *p* = 0.072) was modestly associated with OS (*p* = 0.0718).

**Figure 4:**
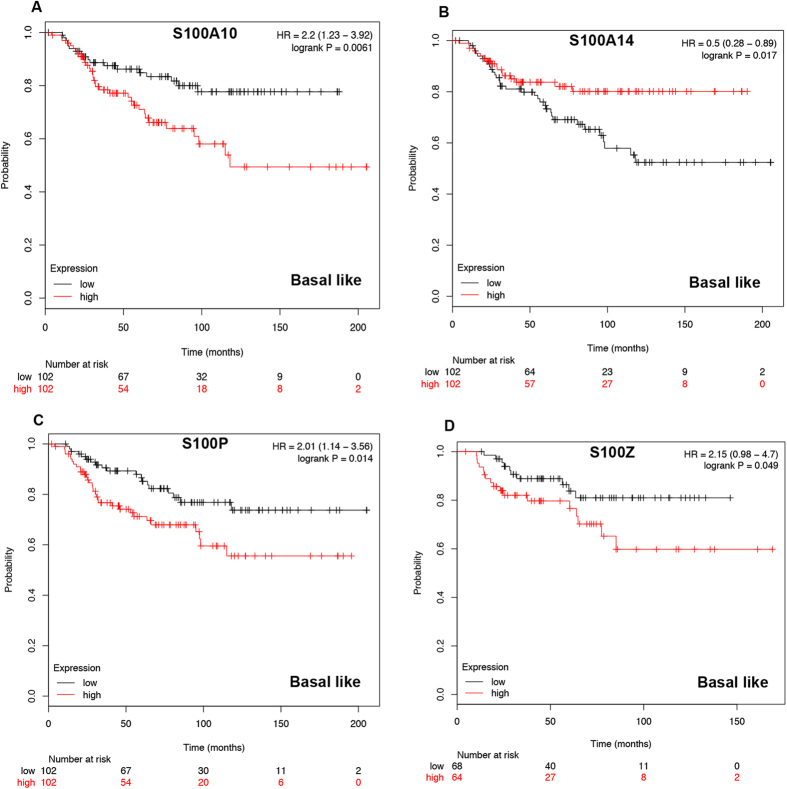
Survival curves of S100A10 (**A**) Affymetrix IDs: 209686_at), S100A14 (**B**) the desired Affymetrix IDs is valid: 218677_at), S100P (**C**) Affymetrix IDs: 204351_at) and S100AZ (**D**) Affymetrix IDs: 1554876_a_at) are plotted for basal-like breast cancer patients (n = 204).

In basal-like breast cancer, mRNA expression of S100A10 (Fig. 4A: HR = 2.2 95%CI: 1.23–3.92, *p* = 0.0061), S100P (Fig. 4C: HR = 2.01, 95%CI: 1.14–3.56, *p* = 0.0139) and S100Z (Fig. 4D: HR = 2.15, 95%CI: 0.98–4.7, *p* = 0.0491) were correlated to worse OS. However, S100A14 (Fig. 4B: HR = 0.5, 95%CI: 0.28–0.89, *p* = 0.0169) was associated with better prognosis. We have observed the survival curves of the rest members of S100 in basal-like breast cancer were not associated with prognosis (Supplement Fig. 5).

### Prognostic values of S100 members in breast cancer patients with different clinicopathological features

Furthermore, we assessed the correlation of the prognostic values of S100 with other clinicopathological features, such as pathological grades, lymph node status and TP53 status. As we can see from Table 1, high mRNA expression of S100A7 (HR = 1.66, 95%CI: 1.04–2.65, *p* = 0.0326), S100A8 (HR = 1.82, 95%CI: 1.13–2.92, p = 0.0117), S100A9 (HR = 2.13, 95%CI: 1.32–3.46, *p* = 0.0016) and S100A12 (HR = 1.65, 95%CI: 1.02–2.65, *p* = 0.0375) were associated with worse OS in grade II breast cancer. S100P high mRNA expression was associated with worse OS in grade I breast cancer patients (HR = 3.46, 95%CI: 1.11–10.8, *p* = 0.0229). None of the S100 mRNA expression was found to be correlated to OS in grade III patients. As from Table 2, S100A8 (HR = 1.87, 95%CI: 1.23–2.84, *p* = 0.0031), S100A9 (HR = 1.85, 95%CI: 1.22–2.82, *p* = 0.0034) and S10010 (HR = 1.94, 95%CI: 1.26–2.96, *p* = 0.0002) were associated with worse survival in lymph node negative breast cancer patients. S100A13 (HR = 0.62, 95%CI: 0.41–0.94, *p* = 0.0240) was associated with better prognosis in lymph node negative breast cancer. Table 3 has shown mRNA expression of S100A8 (HR = 2.57, 95%CI: 1.29–5.14, *p* = 0.0055) and S100P (HR = 2.42, 95%CI: 1.21–4.82, *p* = 0.0095) were correlated to worse OS in wild-p53-type breast cancer. However, S100A4 mRNA elevated expression was associated with better OS in mutant-p53-type breast cancer patients.

## Discussion

In our study, S100A1 and S100A6 were significantly associated with better prognosis, while S100A8, S100A9, S100A11 and S100P were found to be correlated to worse outcome. Dysregulated S100 expression is a common feature in several human cancers. The alterative expression levels of S100 is correlated with progressive disease, but the mechanisms of how individual S100 family members contribute to disease aggression are largely unknown^5^. In breast cancer, only S100A4, S100A7, and the heterodimer S100A8-S100A9 are extensively evaluated. S100A4 potentially enhances tumor metastasis in pre-existing tumorigenic mouse models of breast cancer^29^. The protein expression level of S100A4 was associated with a poor prognosis in stage I and stage II breast cancer^19^. Furthermore, depletion of S100A4 + stromal cells significantly reduced metastatic potential of orthotopic mammary tumor without affecting primary tumor growth^30^. The treatment of anti-S100A4 monoclonal antibody efficiently reduced metastatic burden by suppressing the recruitment of T cells to the primary tumor site^31^. However, in this study, we failed to find any correlation between the mRNA expression of S100A4 and prognosis in luminal A, luminal B, HER2-overexpressing or basal-like breast cancers. Unexpectedly, high mRNA expression of S100A4 was correlated with better OS in mutant-p53-type breast cancer patients, which may indicate the interaction between S100A4 and mutant p53^32^. The results suggested that mRNA level and protein level expression of S100A4 are functional distinct in breast cancer.

S100A7 protein overexpression is associated with high grade and is an independent prognostic indicator in ER-negative invasive ductal carcinomas^17^. S100A7 exerts different functions in breast cancer cells depending on different ER status. In ERα-positive breast cancer cells, S100A7 exhibits tumor suppressor capabilities via downregulation of the β-catenin/TCF4 pathway and enhanced interaction of β-catenin and E-cadherin^9^. Otherwise, S100A7 promotes prosurvival pathways through increased activity of nuclear factor-κB and phospho-Akt and enhances invasive capability by augmenting epidermal growth factor receptor (EGFR) in ERα-negative breast cancer cells^33^,^34^. Here, the high mRNA expression of S100A7 was associated with worse OS in grade II breast cancer, and modestly associated with poor survival for all breast cancer patients (*p* = 0.059).

S100A8 and S100A9 are originally identified in myeloid cells and naturally form a stable heterocomplex state, participating in myeloid cell differentiation^35^. S100A8 and S100A9 protein expression are also frequently detected in poorly differentiated invasive ductal carcinoma of breast cancer^15^. Tumor-induced upregulation of S100A9 protein is suggested to play a critical role in recruitment and accumulation of myeloid-derived suppressor cells(MDSCs) associating with inhibition of dendritic cell differentiation in breast tumor^36^. S100A8 and S100A9 are also critical for the formation of pre-metastatic niche at multiple organ sites^37^. In addition, S100A8 and S100A9 enhance chemoresistance of breast cancer cells by activating the pro-survival ERK1, ERK2 and ribosomal protein S6 kinase β1 pathways^38^. Not unexpectedly, our results confirmed that S100A8 and S100A9 were significantly associated with lower OS for all breast cancer, especially in luminal A type, lymph node negative and grade 2 breast cancer patients.

S100A1 is abundantly expressed in cardiomyocytes, skeletal muscle fibers and neuronal populations and functions as regulation of energy metabolism^39^. But, its role in cancers has rarely explored. The interaction between S100A1 and S100A4 exerts mutually antagonistic effects. Previous study has suggested that S100A1 reduced the anchorage-independent growth, motility and invasion of rat mammary cells by inhibiting biological effects of S100A4^40^. Consistent with this result, our finding showed that the high mRNA expression of S100A1 was significantly correlated with better prognosis in all breast cancer patients.

S100A6 was preferentially expressed in proliferating but not quiescent fibroblasts cells^41^. Increased expression of S100A6 has been reported to be related to the progression and invasive process of several human carcinomas^42^,^43^,^44^,^45^,^46^. Elevated expression of S100A6 protein is an independent prognostic marker in gastric cancer and pancreatic cancer patients^43^,^44^. However, the prognostic role of S100A6 in breast cancer is unknown. According to our results, increased mRNA expression of S100A6 was correlated to better prognosis, especially in luminal A type breast cancer.

S100A11 is considered as a candidate tumor suppressor gene which regulates pathways for Ca^2+^-induced growth arrest in human keratinocytes^47^,^48^. However, S100A11 expression is significantly upregulated in cancers, indicating a progressive role involving in cancer cell growth^49^,^50^,^51^. In breast carcinoma, S100A11 protein has been shown to be expressed in different intrinsic subtypes, and its expression pattern is independent of any clinical parameters^52^. Here, our results supported that increased mRNA expression of S100A11 may indicated worse outcome of breast cancer patients^22^.

S100P was first identified in human placenta^53^, now it is becoming a new potential marker in diagnosing and predicting cancers^21^,^54^,^55^,^56^. The elevated S100P expression is significantly associated with poor survival in operable breast cancer patients^21^,^57^ or in triple-negative breast cancer patients^58^. Recently, Chung *et al*. reported the expression of short form of S100P indicated a worse survival in positive lymph node breast cancer patients^59^. Here, we also confirm the prognostic value of S100P high mRNA expression in breast cancer patients. But high mRNA expression of S100P was not associated with prognosis in positive lymph node breast cancer patients. Furthermore, OS of patients with S100P mRNA abundance was significantly lower in luminal B, grade I or triple-negative breast cancer.

A crosstalk between S100 and estrogen may occur in breast cancer. S100A7 either inhibits or enhances the NF-κB–miR-29b–p53 pathway depending on the ER status^60^. S100A7 mediates differential regulation of actin remodeling and MMP-9 in breast cancer cells depending on the ER status^34^. In addition, estrogen is able to suppress adipogenesis by inhibiting S100A16 in mouse embryonic fibroblasts^61^. HER2 gene amplification occurs in about 20–25% of breast cancers and play an important role in tumor aggression^62^. S100A7 can interact with HER2 signaling through distinct and specific phosphorylation of tyrosine resides of EGFR/HER2, Src and SHP2 in breast cancer cells^63^. S100A14 expression is positively correlated with HER2 expression in breast cancer tissues, and S100A14 can bind to and phosphorylate HER2 in a Ca^2+^-dependent manner and consequently increase cell growth^64^. Furthermore, S100A14 can either promote or inhibit cell motility and invasiveness by regulating MMP2 in a p53-dependent manner^65^. Herein, we observed that S100A14 mRNA expression was correlated to worse OS in luminal B type breast cancer patients, but its increased expression was associated with better OS in TNBC patients, which is not consistent with the results of previous study^22^. The different findings could be due to different patient’s populations, different approaches to determining cut-off points, different follow-up periods and different HER2 or p53 status of breast cancer. In addition, S100P was correlated to worse OS in both basal-like and luminal B type patients. S100A10 and S100Z mRNA expression were associated with lower OS in basal-like breast cancer. S100A5, S100A6, S100A8 and S100A9 were correlated with prognosis in luminal A type breast cancer patients. The expression of S100B mRNA was correlated with better survival in luminal B type breast cancer. The above results suggested that the crosstalk between S100 and estrogen or EGFR/HER2 signaling existed in breast cancer development, and various S100 members interacted with different signaling and exerted different functions.

P53 protein is widely accepted as a tumor suppressor which is capable of inducing cell cycle arrest, senescence and apoptosis. Mutant p53, mostly missense mutations in exons 4–9, possesses a gain-of-function involving in tumorigenesis, invasion and metastasis^66^. Several members of S100 family can directly bind to p53 and inhibit expression and phosphorylation of p53, which promotes stemness of cancer cells, contributes to chemoresistance and leads to cancer progression^67^,^68^,^69^,^70^,^71^. Otherwise, S100A4 may interact with mutant-type p53 and promote its accumulation in cancer cells^32^. Although S100A14 may play a dual role in tumor cells in a p53-dependent manner^65^, increased mRNA expression of S100A14 did not show any relationship with outcome in wild or mutant-p53-type breast cancer. S100A8 and S100P high mRNA expression were correlated to worse OS in wild-p53-type breast cancer. And S100A4 high mRNA expression was associated with better OS in mutant-p53-type breast cancer patients.

Our results indicated that S100A9, S100A11 and S100P were associated with worse outcome in all breast cancer patients according to the Kaplan-Meier survival curves and the log-rank P value based on the database. However, the survival curves of high and low mRNA expression of S100A9, S100A11 and S100P showed an intersection at the time of 200 months, which might indicated some confounding factors existing when doing these analysis. Multivariate analysis by COX regression which can eliminate the confounding factors couldn’t be achieved in this database. Thus the conclusion that S100A9, S100A11 and S100P correlated to worse outcome in breast cancer patients seems plausible, and it’s required to further study the precise prognostic significance of them in breast cancer.

In summary, we assessed the prognostic values of 20 members of S100 mRNA expression in breast cancer patients by KM plotter database. Among them, 6 members were significantly associated with prognosis in breast cancer patients. Further assessment of prognostic values of S100 in breast cancer with different clinical features suggested that different S100 members may interact with different signaling pathways and exerted different functions in breast cancer development.

Our study provides new insights regarding the contribution of S100 members to breast cancer progression and may be of help for the further discovering of S100-target inhibitors for treating breast cancer.

## Additional Information

**How to cite this article**: Zhang, S. *et al*. Distinct prognostic values of S100 mRNA expression in breast cancer. *Sci. Rep.* **7**, 39786; doi: 10.1038/srep39786 (2017).

**Publisher's note:** Springer Nature remains neutral with regard to jurisdictional claims in published maps and institutional affiliations.

## Supplementary Material

Supplemental Information

## Figures and Tables

**Table 1:** Correlation of S100 with different pathological grade status of breast cancer patients.

S100 family	Affymetrix IDs	grades	HR	95%CI	P value
S100A1	205334_at	I	0.88	(0.35, 2.22)	0.7806
		II	0.99	(0.62, 1.57)	0.9668
		III	0.95	(0.64, 1.42)	0.8150
S100A2	204268_at	I	0.53	(0.20, 1.42)	0.1970
		II	1.16	(0.73, 1.84)	0.5261
		III	1.20	(0.80, 1.78)	0.3802
S100A3	206027_at	I	0.76	(0.28, 2.08)	0.5925
		II	0.87	(0.55, 1.39)	0.5724
		III	1.35	(0.90, 2.01)	0.1438
S100A4	203186_s_at	I	1.13	(0.43, 2.96)	0.8091
		II	0.92	(0.58, 1.46)	0.7248
		III	0.72	(0.48,1.08)	0.1159
S100A5	207763_at	I	0.69	(0.26, 1.83)	0.4491
		II	1.43	(0.90, 2.29)	0.1301
		III	1.01	(0.68, 1.50)	0.9778
S100A6	217728_at	I	0.72	(0.28, 1.89)	0.5068
		II	0.64	(0.40, 1.03)	0.0670
		III	0.89	(0.60, 1.33)	0.5716
S100A7	205916_at	I	0.64	(0.22, 1.89)	0.4205
		II	1.66	(1.04, 2.65)	0.0326
		III	1.32	(0.88, 1.97)	0.1746
S100A7A	232170_at	I	—	—	—
		II	3.67	(0.37, 36.09)	0.2341
		III	0.70	(0.35, 1.39)	0.3054
S100A8	202917_s_at	I	1.69	(0.64, 4.51)	0.2860
		II	1.82	(1.13, 2.92)	0.0117
		III	1.01	(0.68, 1.51)	0.9612
S100A9	203535_at	I	1.41	(0.54, 3.68)	0.4817
		II	2.13	(1.32, 3.46)	0.0016
		III	1.32	(0.88, 1.97)	0.1728
S100A10	200872_at	I	1.50	(0.57, 3.90)	0.4068
		II	0.85	(0.53, 1.35)	0.4918
		III	1.21	(0.81, 1.80)	0.3573
S100A11	200660_at	I	2.62	(0.91, 7.56)	0.0652
		II	1.37	(0.86, 2.18)	0.1864
		III	1.15	(0.77, 1.72)	0.4830
S100A12	205863_at	I	1.62	(0.63, 4.14)	0.3092
		II	1.65	(1.02, 2.65)	0.0375
		III	1.14	(0.76, 1.70)	0.5365
S100A13	202598_at	I	0.86	(0.34, 2.17)	0.7448
		II	0.79	(0.50, 1.26)	0.3202
		III	1.07	(0.71, 1.59)	0.7583
S100A14	218677_at	I	1.64	(0.61, 4.35)	0.3200
		II	0.84	(0.53, 1.33)	0.4592
		III	0.92	(0.62, 1.37)	0.6784
S100A16	218677_at	I	—	—	—
		II	0.33	(0.03, 3.19)	0.3143
		III	1.08	(0.55, 2.11)	0.8278
S100B	209686_at	I	1.67	(0.63, 4.41)	0.2989
		II	0.76	(0.48, 1.22)	0.2591
		III	0.74	(0.49, 1.11)	0.1400
S100P	204351_at	I	3.46	(1.11, 10.8)	0.0229
		II	1.48	(0.92, 2.35)	0.1006
		III	1.24	(0.83, 1.84)	0.2993
S100Z	204351_at	I	—	—	—
		II	3.72	(0.39, 35.92)	0.2228
		III	1.49	(0.76, 2.93)	0.2414
S100G	207885_at	I	0.71	(0.26, 1.94)	0.5079
		II	1.10	(0.69, 1.75)	0.6879
		III	1.04	(0.70, 1.55)	0.8482

**Table 2:** Correlation of S100 members with different lymph node status of breast cancer patient.

S100 family	Affymetrix IDs	Lymph node status	HR	95%CI	P value
S100A1	205334_at	negative	0.66	(0.44, 1.01)	0.0518
		positive	1.38	(0.83, 2.29)	0.2098
S100A2	204268_at	negative	1.02	(0.68, 1.54)	0.9248
		positive	1.30	(0.79, 2.15)	0.2973
S100A3	206027_at	negative	0.87	(0.57, 1.32)	0.5093
		positive	1.58	(0.95, 2.63)	0.0737
S100A4	203186_s_at	negative	1.03	(0.68, 1.56)	0.8766
		positive	0.83	(0.51, 1.37)	0.4768
S100A5	207763_at	negative	0.87	(0.58, 1.32)	0.5258
		positive	1.32	(0.80, 2.19)	0.2728
S100A6	217728_at	negative	0.68	(0.45, 1.04)	0.0758
		positive	1.12	(0.68, 1.84)	0.6640
S100A7	205916_at	negative	1.40	(0.93, 2.12)	0.1097
		positive	1.02	(0.62, 1.67)	0.9528
S100A7A	232170_at	negative	1.01	(0.33, 3.14)	0.9827
		positive	0.64	(0.30, 1.37)	0.2451
S100A8	202917_s_at	negative	1.87	(1.23, 2.84)	0.0031
		positive	0.79	(0.47, 1.31)	0.3598
S100A9	203535_at	negative	1.85	(1.22, 2.82)	0.0034
		positive	1.10	(0.66, 1.82)	0.7075
S100A10	200872_at	negative	1.94	(1.26, 2.96)	0.0002
		positive	0.88	(0.53, 1.46)	0.6149
S100A11	200660_at	negative	1.42	(0.93, 2.14)	0.0990
		positive	0.83	(0.50, 1.37)	0.4579
S100A12	205863_at	negative	1.29	(0.91, 2.12)	0.1243
		positive	1.28	(0.78, 2.11)	0.3334
S100A13	202598_at	negative	0.62	(0.41, 0.94)	0.0240
		positive	1.38	(0.82, 2.32)	0.2198
S100A14	218677_at	negative	0.74	(0.49, 1.12)	0.1534
		positive	1.48	(0.89, 2.46)	0.1271
S100A16	227998_at	negative	1.16	(0.37, 3.62)	0.7941
		positive	0.96	(0.46, 2.01)	0.9092
S100B	209686_at	negative	1.13	(0.75, 1.70)	0.5749
		positive	0.65	(0.39, 1.10)	0.1039
S100P	204351_at	negative	1.47	(0.97, 2.23)	0.0666
		positive	1.00	(0.61, 1.64)	0.9900
S100Z	1554876_a_at	negative	0.93	(0.30, 2.88)	0.8965
		positive	1.59	(0.75, 3.35)	0.2214
S100G	207885_at	negative	1.00	(0.66, 1.50)	0.9822
		positive	1.12	(0.68, 1.85)	0.6500

**Table 3:** Correlation of S100 members with different p53 status of breast cancer patients.

S100 family	Affymetrix IDs	p53	HR	95%CI	P value
S100A1	205334_at	mutant	1.59	(0.72, 3.48)	0.2452
		wild	1.07	(0.56, 2.04)	0.8319
S100A2	204268_at	mutant	0.98	(0.46, 2.08)	0.9554
		wild	0.99	(0.52, 1.88)	0.9697
S100A3	206027_at	mutant	0.91	(0.42, 1.97)	0.8206
		wild	0.85	(0.45, 1.63)	0.6338
S100A4	203186_s_at	mutant	0.36	(0.16, 0.83)	0.0126
		wild	0.91	(0.48, 1.74)	0.7858
S100A5	207763_at	mutant	0.7	(0.32, 1.51)	0.3582
		wild	1.27	(0.66, 2.43)	0.4684
S100A6	217728_at	mutant	0.96	(0.45, 2.05)	0.9134
		wild	0.93	(0.49, 1.78)	0.8288
S100A7	205916_at	mutant	0.59	(0.27, 1.28)	0.1754
		wild	1.7	(0.88, 3.29)	0.1102
S100A7A	232170_at	mutant	1.08	(0.28, 4.06)	0.9148
		wild	—	—	—
S100A8	202917_s_at	mutant	0.58	(0.27, 1.28)	0.1755
		wild	2.57	(1.29, 5.14)	0.0055
S100A9	203535_at	mutant	0.61	(0.28, 1.34)	0.2109
		wild	1.9	(0.97, 3.69)	0.0561
S100A10	200872_at	mutant	1.16	(0.54, 2.51)	0.7045
		wild	0.78	(0.41, 1.49)	0.4501
S100A11	200660_at	mutant	0.57	(0.26, 1.23)	0.1440
		wild	1.6	(0.83, 3.08)	0.1569
S100A12	205863_at	mutant	1.16	(0.53, 2.55)	0.7072
		wild	1.62	(0.84, 3.13)	0.1449
S100A13	202598_at	mutant	1.51	(0.69, 3.33)	0.3005
		wild	1.21	(0.64, 2.32)	0.5550
S100A14	218677_at	mutant	1.19	(0.56, 2.54)	0.6462
		wild	1.48	(0.77, 2.86)	0.2357
S100A16	227998_at	mutant	0.78	(0.21, 2.93)	0.7143
		wild	—	—	—
S100B	209686_at	mutant	0.54	(0.24, 1.21)	0.1303
		wild	0.58	(0.30, 1.14)	0.1109
S100P	204351_at	mutant	1.06	(0.50, 2.26)	0.8797
		wild	2.42	(1.21, 4.82)	0.0095
S100Z	1554876_a_at	mutant	1.33	(0.36, 4.94)	0.6732
		wild	—	—	—
S100G	207885_at	mutant	1.38	(0.64, 2.99)	0.4158
		wild	0.91	(0.48, 1.74)	0.7708
