# Supplementary material for: Distinct prognostic values of S100 mRNA expression in breast cancer
Source: Sci Rep. 2017 Jan 4;7:39786. doi: 10.1038/srep39786 (PMC5209742; doi:10.1038/srep39786)

Distinct prognostic values of S100 mRNA expression in breast cancer

Shizhen Zhang[1,2]*, Zhen Wang[1,2]*, Weiwei Liu[3]*, Rui Lei[4], Jinlan Shan[1,2], Ling Li[5], Xiaochen Wang[1,2#]

1Department of Surgical Oncology, 2Cancer Institute (Key Laboratory of Cancer Prevention & Intervention, National Ministry of Education, Provincial Key Laboratory of Molecular Biology in Medical Sciences) and 3Department of Laboratory Medicine, Second Affiliated Hospital, Zhejiang University School of Medicine, Hangzhou, Zhejiang, China; 4Department of Plastic Surgery, First Affiliated Hospital, Zhejiang University School of Medicine, Hangzhou, China. 5Division of Hematopoietic Stem Cell and Leukemia Research, Beckman Research Institute, City of Hope National Medical Center, Duarte, CA, USA

Supplemental figure legends

Supplemental figure 1: A-L: Survival curves of S100A2, S100A3, S100A5, S100A7A, S100A10, S100A12, S100A13, S100A14, S100A16, S100B, S100Z and S100G are plotted for all patients.

Supplemental figure 2: A-N: Survival curves of S100A3, S100A4, S100A7, S100A7A, S100A10, S100A11, S100A12, S100A13, S100A14, S100A16, S100B, S100P, S100Z and S100G are plotted for luminal A type breast cancer patients.

Supplemental figure 3: A-Q: Survival curves of S100A1, S100A2, S100A3, S100A4, S100A5, S100A6, S100A7, S100A7A, S100A9, S100A10, S100A11, S100A12, S100A13, S100A14, S100A16, S100Z and S100G are plotted for luminal B type breast cancer patients.

Supplemental figure 4: None of S100 family members mRNA expression indicated significant relationship with OS for HER2-overexpressing type breast cancer patients. A-T: Survival curves of S100A1, S100A2, S100A3, S100A4, S100A5, S100A6, S100A7, S100A7A, S100A8, S100A9, S100A10, S100A11, S100A12, S100A13, S100A14, S100A16, S100B, S100P, S100Z and S100G are plotted.

Supplemental figure 5: A-P: Survival curves of S100A1, S100A2, S100A3, S100A4, S100A5, S100A6, S100A7, S100A7A, S100A8, S100A9, S100A10, S100A11, S100A12, S100A13, S100A16, S100B and S100G are plotted for basal-like type breast cancer patients.

Supplemental Figure 1

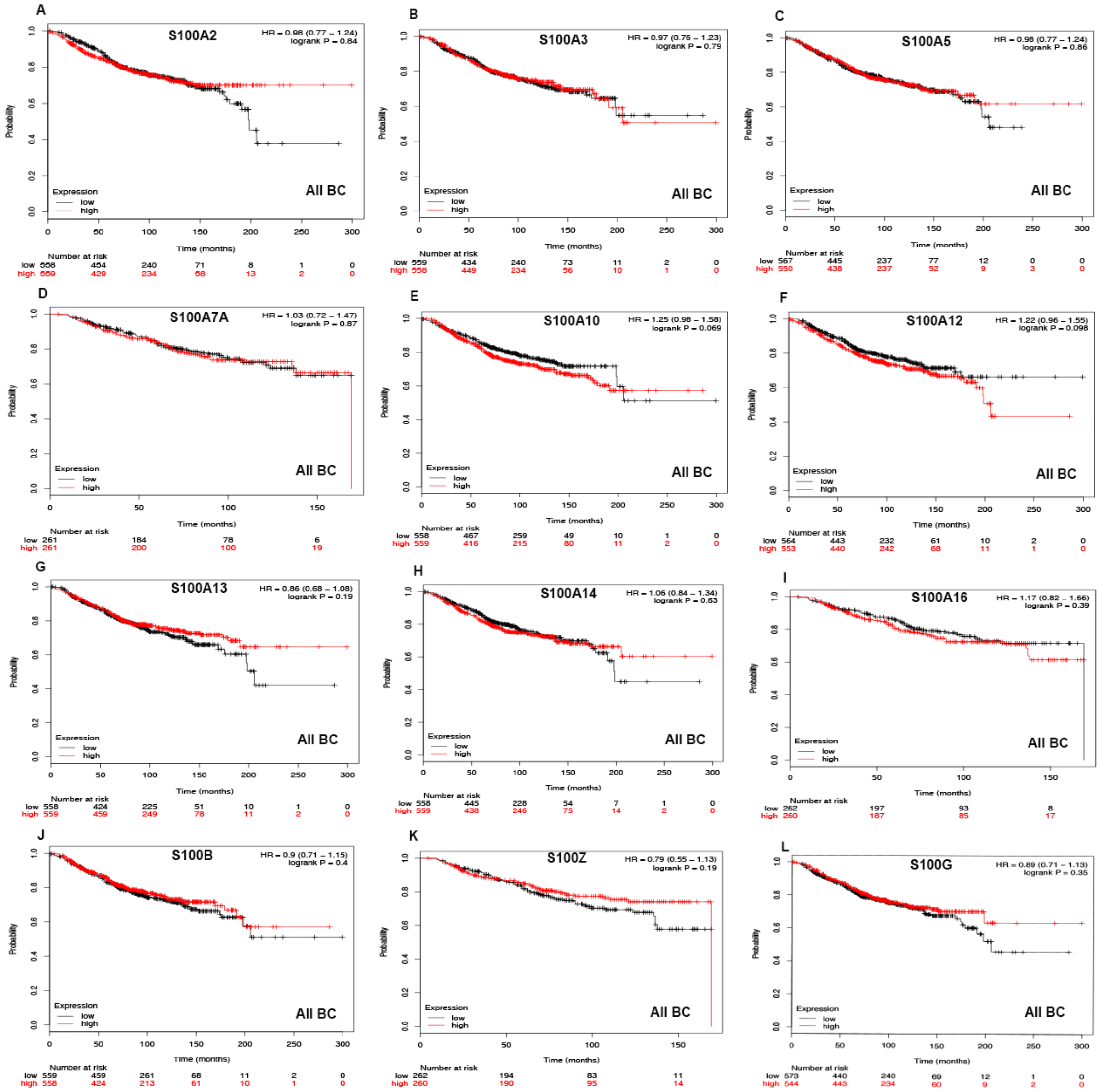

Supplemental Figure 2

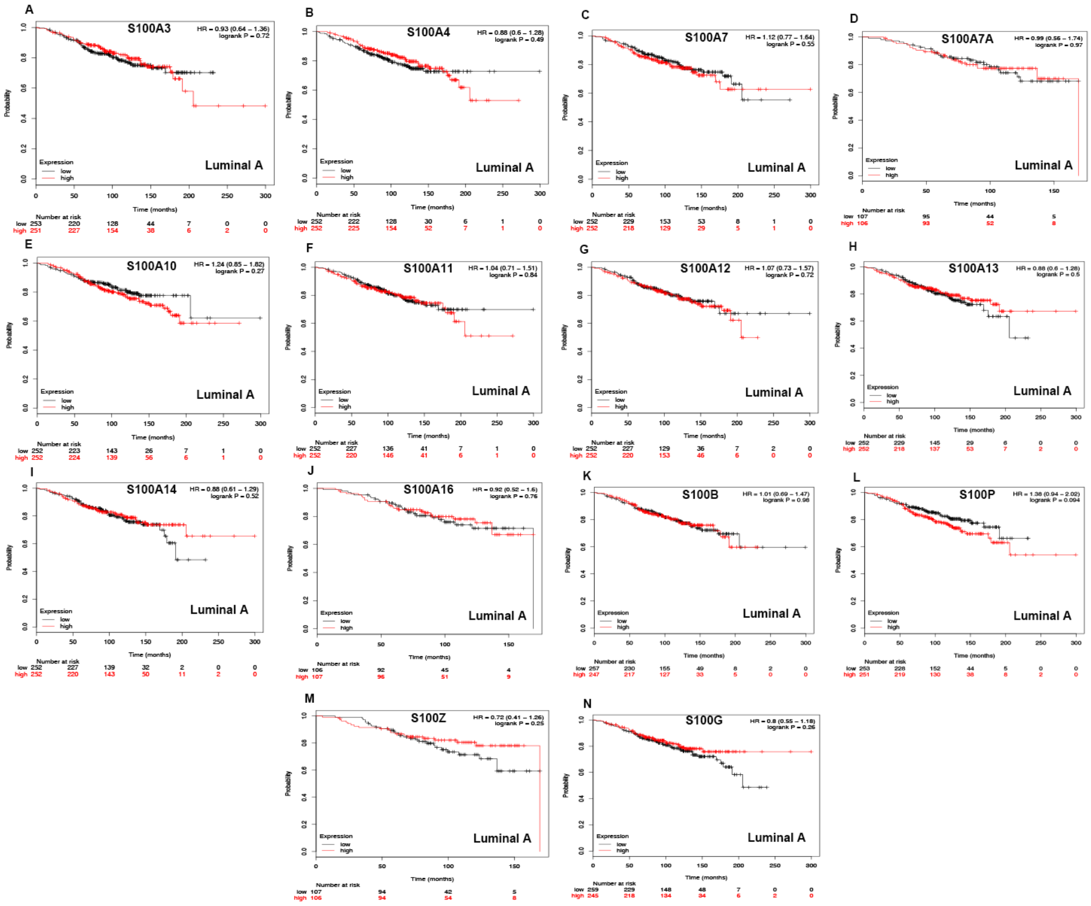

Supplemental Figure 3

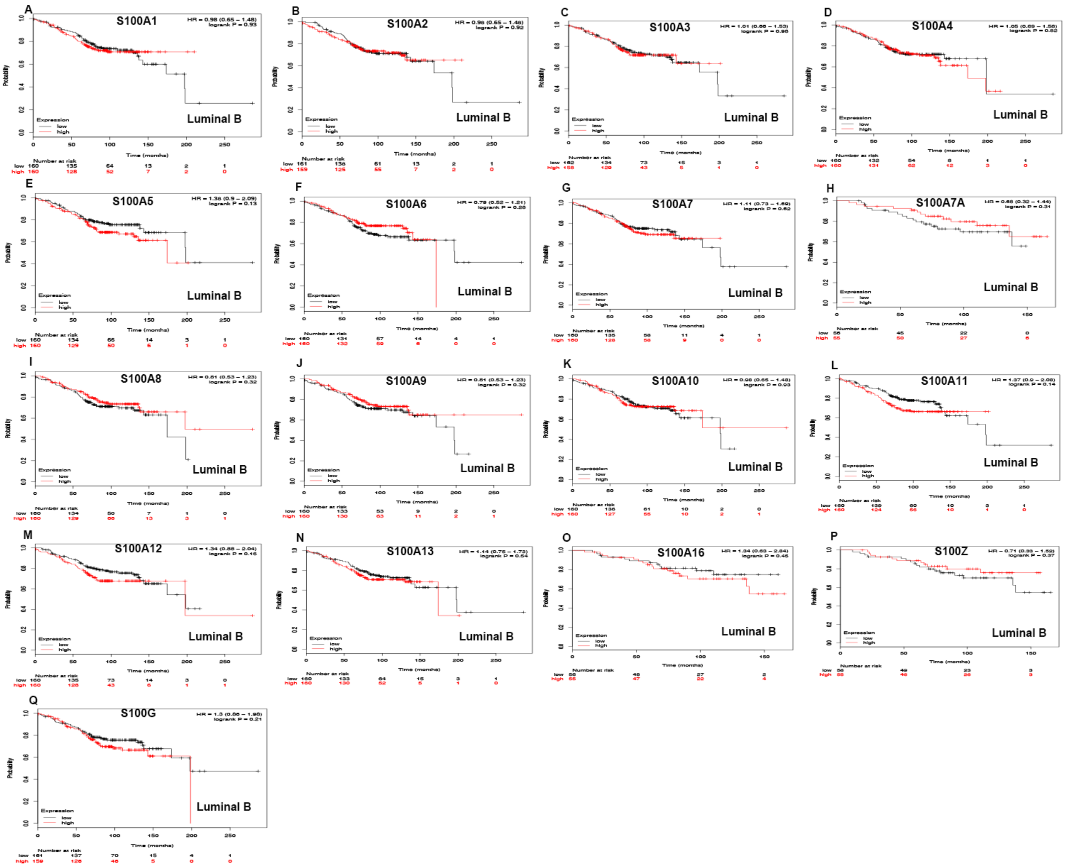

Supplemental Figure 4

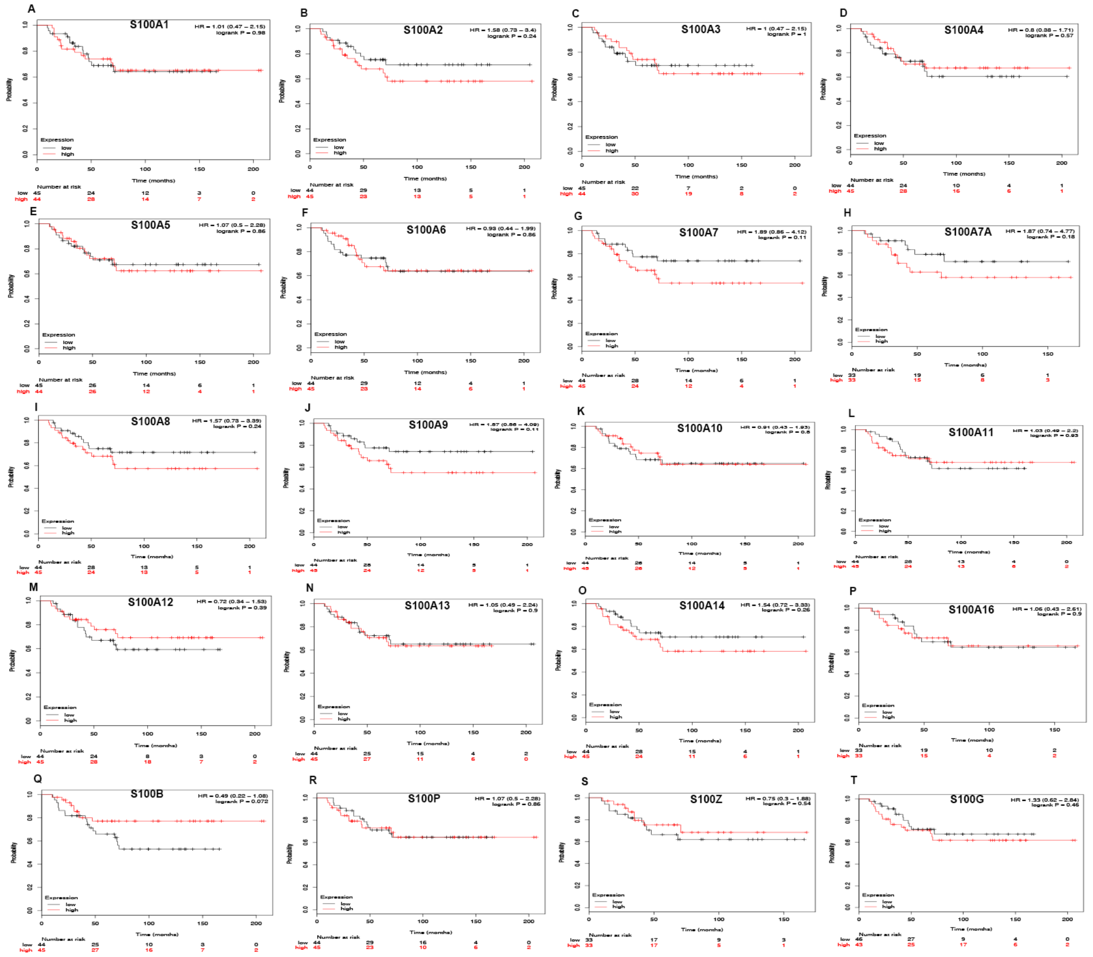

Supplemental Figure 5

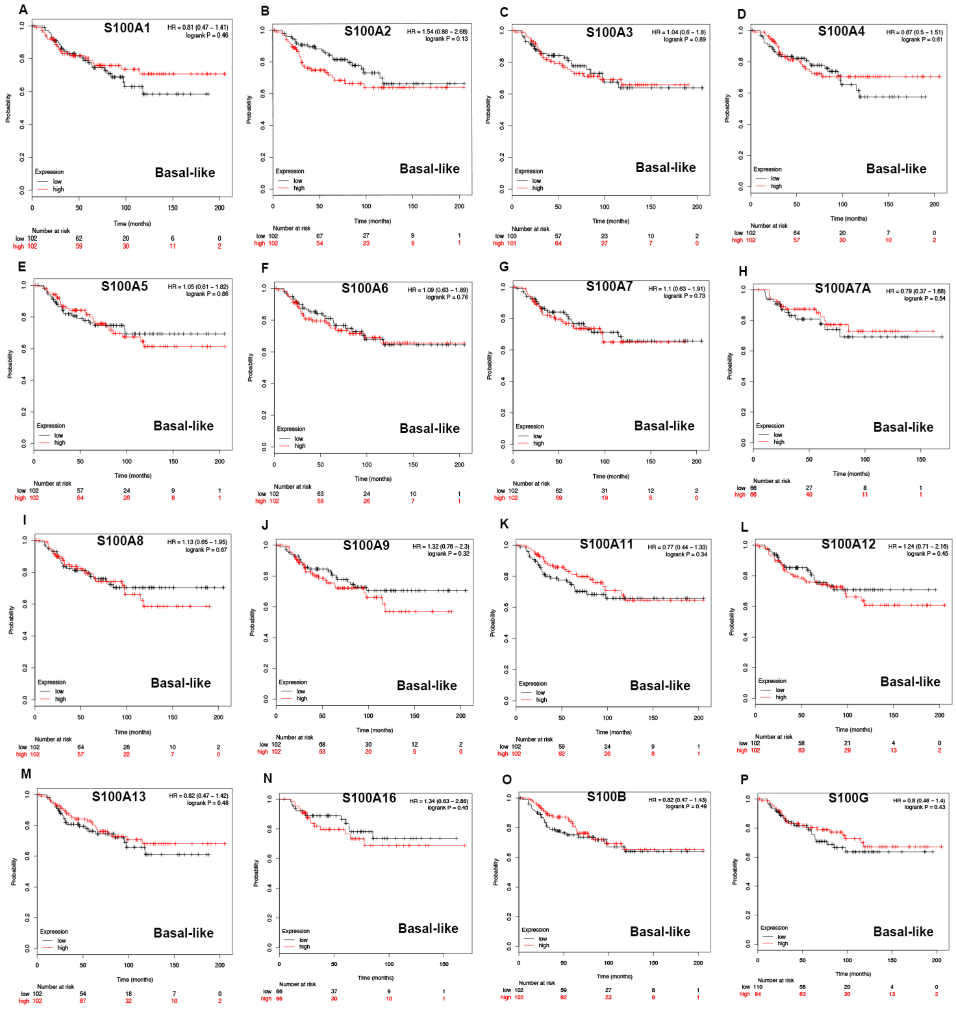

Supplement: Supplemental Information [file srep39786-s1.pdf]
